# Diagnostic utility of contrast-enhanced ultrasound endoscopy in a case of hemosuccus pancreaticus　

**DOI:** 10.1055/a-2307-5744

**Published:** 2024-05-07

**Authors:** Kiyokuni Tanabe, Atsushi Kanno, Eriko Ikeda, Kozue Ando, Kensuke Yokoyama, Hironori Yamamoto

**Affiliations:** 1 12838 Department of Medicine, Division of Gastroenterology, Jichi Medical University, Shimotsuke, Japan

Hemosuccus pancreaticus is a rare condition characterized by bleeding from an intrapancreatic pseudocyst or ruptured peripancreatic aneurysm emptying from the main pancreatic duct (MPD) into the gastrointestinal tract via the duodenal papilla 1 . Contrast-enhanced computed tomography (CE-CT) is the primary diagnostic modality for hemosuccus pancreaticus. However, the indication for CE-CT should be carefully evaluated in individuals with renal dysfunction or an allergy to contrast media 2 . We describe a case where contrast-enhanced endoscopic ultrasound (CE-EUS) was useful in diagnosing hemosuccus pancreaticus ( Video 1 ).

Contrast-enhanced endoscopic ultrasound revealed a bleeding cystic area with debris indicative of hemosuccus pancreaticus.Video 1

A 64-year-old man under follow-up for chronic pancreatitis presented with tarry stools. Clinical examination revealed anemia, renal failure (creatinine 2.5 U/L), and elevated pancreatic enzyme levels (amylase 324 IU/L; lipase 473 IU/L). CE-CT was difficult to perform due to renal dysfunction. Endoscopic findings revealed bleeding from the duodenal papilla. In EUS, high-echoic debris suggestive of hemorrhage was observed in the MPD. However, B-mode findings alone could not identify the hemorrhaging areas in the pancreatic parenchyma ( Fig. 1 ). CE-EUS with Sonazoid (GE Healthcare, Oslo, Norway) revealed a pancreatic cystic lesion with debris ( Fig. 2 ), the suspected bleeding point of the hemosuccus pancreaticus. However, the cystic lesion was obscured on plain CT ( Fig. 3 ). After adequate hydration, CE-CT revealed contact between the anterior superior pancreaticoduodenal artery (ASPDA) and the cystic lesion. Angiography revealed irregular caliber changes in the ASPDA, confirming it as the bleeding point. Next, coil embolization was performed ( Fig. 4 ), which subsequently improved stool quality and anemia.

**Fig. 1:**
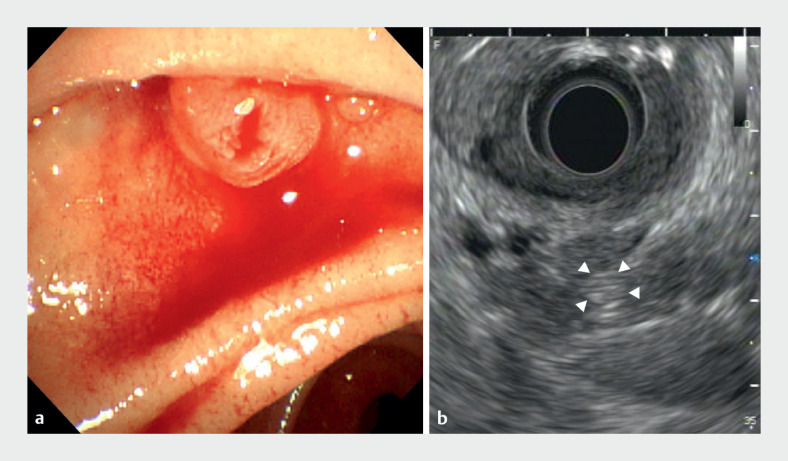
Endoscopic ultrasound (EUS) images. **a** Endoscopic observation during EUS showed bleeding from the duodenal papilla. **b** B-mode imaging demonstrated highly echogenic debris suggestive of a hematoma in the main pancreatic duct (arrowheads).

**Fig. 2:**
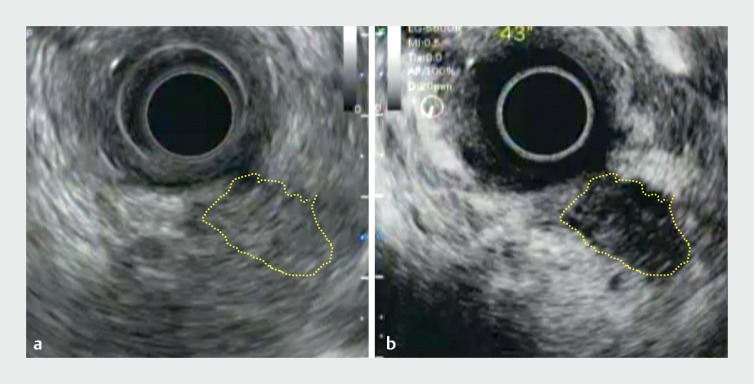
Endoscopic ultrasound images. **a** B-mode observation revealed no lesions in the pancreatic parenchyma. **b** Contrast-enhanced endoscopic ultrasound with Sonazoid (GE Healthcare, Oslo, Norway) showed a pancreatic cystic lesion with debris (yellow dotted line).

**Fig. 3:**
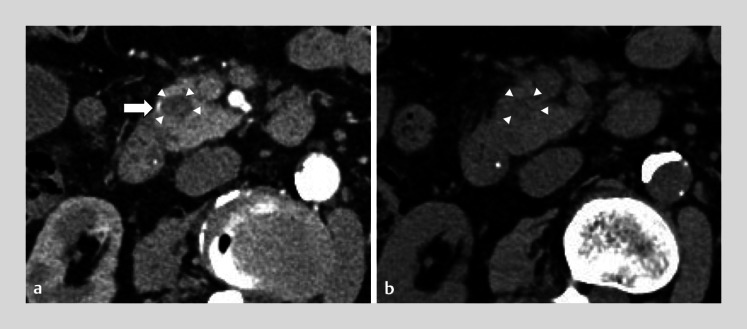
Computed tomography (CT) images. **a** Contrast-enhanced CT revealed the anterior superior pancreaticoduodenal artery (arrow) in contact with the cystic lesion with internal hematoma (arrowheads). **b** The cystic lesion was obscured by plain CT (arrowheads).

**Fig. 4:**
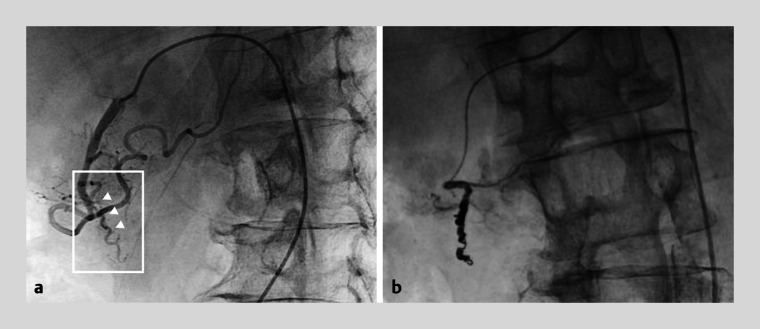
Angiography images. **a** Angiography showed irregular caliber changes in the anterior superior pancreaticoduodenal artery (arrowheads). **b** Coil embolization was performed on the vessel causing the bleeding.

Diagnosing hemosuccus pancreaticus using B-mode EUS or plain CT is challenging because of poor visualization of the hemorrhage area in the pancreatic parenchyma. CE-EUS is valuable in identifying the small arterial pseudoaneurysm with a fistula to the MPD 3 4 . CE-EUS can visualize the area of hemosuccus pancreaticus in patients who cannot undergo CE-CT due to renal dysfunction or other reasons.

Endoscopy_UCTN_Code_TTT_1AS_2AG

## Notes

Endoscopy E-Videos https://eref.thieme.de/e-videos
*E-Videos* is an open access online section of the journal *Endoscopy* , reporting on interesting cases and new techniques in gastroenterological endoscopy. All papers include a high-quality video and are published with a Creative Commons CC-BY license. Endoscopy E-Videos qualify for HINARI discounts and waivers and eligibility is automatically checked during the submission process. We grant 100% waivers to articles whose corresponding authors are based in Group A countries and 50% waivers to those who are based in Group B countries as classified by Research4Life (see: https://www.research4life.org/access/eligibility/ ). This section has its own submission website at https://mc.manuscriptcentral.com/e-videos .
